# HIV and HCV co-infection in patients on antiretroviral therapy: a case study of selected health facilities in Ilorin, Nigeria

**DOI:** 10.1186/s12879-026-12658-3

**Published:** 2026-01-22

**Authors:** Felicia E. Williams, Jane-Frances I. John-Benson, Winifred D. Giwa, David U. Adje, Louis O. Odeigah, Chinonyerem O. Iheanacho, Isabel N. Aika

**Affiliations:** 1 Department of Clinical Pharmacy and Pharmacy Practice, Faculty of Pharmaceutical Sciences, University of Ilorin, Ilorin, Nigeria; 2 Medplus Pharmacy, 45A Ogudu Road, Lagos, Nigeria; 3 Duchess International Hospital, GRA, Ikeja, Lagos, Nigeria; 4 Department of Clinical Pharmacy and Pharmacy Administration, Faculty of Pharmacy, Delta State University, Abraka, Nigeria; 5 Department of Family Medicine, Faculty of Clinical Sciences, College of Medicine & Health Sciences, Afe Babalola University, Ado-Ekiti, Nigeria; 6 Department of Clinical Pharmacy and Public health, Faculty of Pharmacy, University of Calabar, Calabar, Nigeria; 7 Department of Clinical Pharmacy and Pharmacy Practice, Faculty of Pharmacy, University of Benin, Benin City, Nigeria

**Keywords:** Antiretroviral therapy, HIV/HCV co-infection, HCV seroprevalence, National HIV Treatment Guidelines in Nigeria, People living with HIV

## Abstract

**Background:**

Human Immunodeficiency Virus (HIV)/Hepatitis C Virus (HCV) co-infection is an important healthcare challenge partly due to adverse prognosis of the dual disease burden. This study assessed the prevalence of HIV/HCV co-infection and associated factors in patients on antiretroviral therapy (ART) in public secondary care hospitals in Ilorin, Nigeria.

**Methods:**

This cross-sectional study was conducted in two public secondary care hospitals that provide ART to people living with HIV (PLHIV) in Ilorin, Nigeria. Based on Fisher’s formula, 303 study participants (SPs) were recruited using convenient sampling. Eligibility was based on consent, aged between 18 and 70 years, and receipt of ART for more than 6 months. All SPs received rapid diagnostic screening for HCV antibodies. Clinical and treatment data were obtained from their medical records. Descriptive and inferential (Fisher’s Exact Test) statistics were used to analyse the data with statistical significance set at *p* < 0.05.

**Results:**

Median age of SPs was 40 years, (interquartile range = 48 − 35), most were married (260/303 [85.8%]) and females (238/303 [78.5%]). Prevalence of HIV/HCV co-infection was 2.3% ([7/303] 95% CI, 0.9–4.7%). All co-infected SPs were married females and on Tenofovir/Lamivudine/Dolutegravir. Co-infection was higher in age group ≤ 40 years (4/7 [57.1%]) although not significantly associated with age but associated with viral load (*p* < 0.001).

**Conclusion:**

We found that HIV/HCV co-infection disproportionately affected young married women in this cohort of PLHIV on ART. Implementation of National HIV Treatment guidelines in Nigeria on routine HCV screening among PLHIV for early detection and appropriate management should be prioritized.

**Clinica trial:**

Not applicable.

## Introduction

Human Immunodeficiency Virus (HIV)/Hepatitis C Virus (HCV) co-infection poses a critical public health challenge due to the disease burden, treatment complications and greater stress in healthcare systems in comparison to HIV or HCV mono-infection [1]. A 2016 global review on the prevalence and burden of HCV co-infection in people living with HIV (PLHIV) stated that “due to shared routes of transmission, 2.3 million (6.2%) of PLHIV are estimated to be co-infected with HCV globally” [2, 3]. In 2022, there were 39.0 million PLHIV, 1.3 million people became newly infected and 630,000 AIDs-related deaths globally [4]. Conversely, 50 million people had HCV, incidence of 1 million people while HCV-related mortality was 240,000 people globally [5].

Regarding sub–Saharan Africa, a meta-analysis of 213 studies involving approximately 1.2 million people in 33 countries, found a HIV/HCV prevalence rate of 6%. The rates varied from nearly 4.5% in Southeast Africa to 7% in West Africa [6]. In Nigeria, a national hospital-based prospective study reported 0.14% HIV/HCV co-infection prevalence in 2025 [7]. Another hospital-based study reported 10.5% HIV/HCV co-infection prevalence in Bauchi State in 2024 [8] while an earlier study reported 0% HIV/HCV co-infection prevalence in Lagos in 2019 [9]. Another study in Lagos reported 0.5% HIV/HCV co-infection prevalence [10]. Furthermore, 23.2% HIV/HCV co-infection prevalence was reported in a teaching hospital [11]. However, a study in University of Ilorin Teaching Hospital, Ilorin, Kwara state, reported 0% HIV/HCV co-infection prevalence in 2008 [12].

HIV/HCV co-infection is associated with higher rates of end-stage liver disease-related deaths when compared with HCV mono-infection, suggesting that HIV infection aggravates the progression of liver damage in HIV/HCV co-infected patients [13]. Liver disease related mortality secondary to cirrhosis, liver failure and hepatocellular carcinoma (HCC) were responsible for 20–25% of all causes of mortality of HIV/HCV co-infected people [14]. HIV/HCV co-infected patients have higher propensity of experiencing hepatotoxicity from antiretroviral therapy (ART), show reduced CD4-cell recovery while on ART and may suffer greater damage from immune activation due to HIV replication than HIV mono-infected patients [15]. This immune activation results from immune dysfunction and cytokine production, causing enhanced HIV and HCV replication and lower T-cell counts [16]. In the absence of routine HCV screening, patients and healthcare providers may remain unaware of HCV infection until the onset of cirrhosis and liver failure. This is particularly of concern as HCV infection progresses more rapidly in PLHIV. In addition to other preventive measures to HIV/HCV co-infection such as refraining from sharing needles and personal items that involve blood transmission and condom use for sexual activities, conducting screening for HCV infection periodically in PLHIV is an evidence-based intervention for early detection [17]. This can result in effective management of both disease conditions. Therefore, this study will contribute to the existing literature on HIV/HCV co-infection which is crucial because of its significant impact on disease progression, treatment challenges, and public health implications. Moreover, the National HIV Treatment guidelines in Nigeria, now strongly recommend screening for HCV among PLHIV initiating ART [18]. Adherence to this recommendation will prevent exposure of HIV/HCV co-infected PLHIV to ART-related hepatotoxicity and other HCV associated disorders. Previous studies indicate variations in HIV/HCV co-infection prevalence in different communities in Nigeria at different times. HIV/HCV co-infection is still a public health challenge. A study conducted in Ilorin over 10 years ago reported 0% HIV/HCV co-infection prevalence. Paucity of recent epidemiological information on HIV/HCV co-infection prevalence in Ilorin, necessitated this study. It assessed the prevalence of HIV/HCV co-infection and associated factors in PLHIV in Ilorin. The findings are expected to contribute to the body of knowledge on the prevalence HIV/HCV co-infection in Nigeria. This will also highlight the need for the implementation of National HIV Treatment guidelines in Nigeria on HCV screening among PLHIV with resultant improvement on the quality of clinical care for PLHIV.

## Methods

### Study setting

This study was conducted at two public secondary care hospitals that provide care to PLHIV in Ilorin, Kwara State, Nigeria. There were four (4) secondary healthcare facilities that provide care to PLHIV in Ilorin as at the time of the study. One was undergoing renovation; another was used for the pretest of the study protocol while the study was conducted in the remaining two. The hospitals were: Children Specialist Hospital (CH) and Civil Service Hospital (CS). Kwara State is located in the North Central geopolitical zone of Nigeria and is the gateway between the Southern and Northern parts of Nigeria. Ilorin is the state capital of Kwara State with a population of 854,737 as at 2006 census, and an annual growth rate of 2.53% which resulted in a population projection of 974,000 in 2021 [19–21]. Ilorin comprises three local government areas (LGAs): Ilorin East LGA, Ilorin South LGA and Ilorin West LGA. The predominant indigenous tribes in Ilorin are Yorubas and Hausa-Fulanis [21].

### Study design and study population

This was a cross-sectional study. Rapid diagnostic screening for HCV antibodies was conducted for eligible participants. All adult PLHIV visiting the health facilities within the study period (January 2021 to May 2021) who consented to participate were eligible.

### Inclusion and exclusion criteria

PLHIV aged 18 to 70 years who had received ART for more than 6 months and provided informed consent were included. PLHIV who were too ill or had behavioural disorder that might impair their ability to give informed consent were excluded from the study.

### Sample size calculation

The total population of PLHIV on ART from the two secondary care hospitals was 1483 as at February 2021. The sample size of 303 was determined through use of Fisher’s statistical formula [22] and 55% prevalence of PLHIV on ART in Nigeria [23]. The level of confidence and margin of error for the minimum sample size were 95% and 5% respectively. The sample size was proportionally allocated to each hospital based on 950 and 533 PLHIV that were assessing care in Civil Service Hospital (CS) and Children Specialist Hospital (CH) respectively as at time of the study. Civil Service Hospital (CS) had 194 while Children Specialist Hospital (CH) had 109 of the sample size.

### Sampling

Eligible PLHIV were recruited for the study using convenient sampling. Systematic sampling could not be used due to the low patient hospital attendance because of post-COVID-19 pandemic lock down in Nigeria. All eligible PLHIV who attended general outpatient department clinic were recruited at the Pharmacy Department to ensure adherence to the inclusion and exclusion criteria. Blood samples of eligible PLHIV were obtained and tested at the Clinical Laboratory Department by a Clinical Laboratory Technician. Medical records of eligible PLHIV were retrieved from the Medical Records Department and accessed at the Pharmacy Department on their non-clinic days.

### Data collection tool and procedure

Data extraction forms adapted from previous studies [24, 25] were used to obtain the patients’ socio-demographic, clinical and treatment variables. Also, detection of the presence of HCV antibodies was achieved using Micropoint Rapid Anti-HCV Test (Whole Blood/Serum/Plasma) by InTec Products Inc., China. Whole blood sample obtained from simple fingerstick was used for the HCV antibodies screening. Obtaining this sample was less invasive, requiring less equipment and training as compared to use of serum or plasma samples. This sampling method was also suitable for our relatively large sample size.

### Data analysis

The Statistical Package for Social Sciences (SPSS) version 25.00 was used for data entry and data analyses. The main study outcomes were prevalence of HCV in the study participants. Descriptive statistical analyses were carried out to generate frequency, distribution proportions, central tendencies and dispersions of the variables. Fisher’s Exact Test was performed to explore associations between HIV/HCV co-infection and demographic, clinical and treatment variables. The statistical significance was set at *p* < 0.05.

## Ethical consideration

Ethical approval was obtained from the Kwara State Ministry of Health, Ethical Review Committee with reference number MOH/KS/EU/777/475. Cooperation of relevant heads of departments at the study sites was sought and obtained. Written informed consent was obtained from all study participants (SPs) before inclusion in the study. The study was conducted in accordance with the “Declaration of Helsinki”, 2013 revised edition [26]. Confidentiality of data was ensured.

## Results

### Demographic characteristics of the study participants

The median age of SPs was 40.1 years, (interquartile range = 13 [48 − 35]) and most were females (238/303 [78.5%]). More than half of the respondents were secondary school certificate holders (166/303 [54.8%]) as shown in Table 1.

**Table 1:** Demographic characteristics of PLHIV on ART enrolled in the study (*n* = 303)

Variables	Responses	Frequency	(%)
Age (years)	≤ 20	12	4.0
	21 – 40	141	46.5
	41 – 60	125	41.3
	≥ 61	25	8.2
Median Age (IQR) = 40 (48 − 35) years			
Gender	Male	65	21.5
	Female	238	78.5
Educational Qualification	Secondary	166	54.8
	Undergraduate	93	30.7
	Graduate	2	0.6
	Others	42	13.9
Marital Status	Single	32	10.6
	Married	260	85.8
	Divorced	11	3.6
Number of Wives (*n* = 44)	1	24	54.5
	2	18	40.9
	3	2	4.6
Religion	Islam	199	65.7
	Christianity	104	34.3

IQR = Interquartile range

### Clinical variables of the study participants

More than half of the SPs (166/303 [51.1%]) had been diagnosed of HIV for 6–10 years in comparison with those diagnosed 1–5 and 11–15 years prior to this study. Most SPs (279/303 [92.1%]) had viral load suppression (Table 2). All SPs were in stage I WHO HIV clinical stage at entry into PLHIV care and had no history of hepatitis C infection.

**Table 2:** Clinical characteristics of PLHIV on ART enrolled in the study (*n* = 303)

Variables	Responses	Frequency	(%)
Weight (Kg)	≤ 30	2	0.7
	31 – 60	119	39.2
	61 – 90	146	48.2
	91–120	13	4.3
	Missing	23	7.6
Median weight (IQR) = 62 (74.75-54) kg			
Height (cm)	≤ 80	1	0.3
	81–120	1	0.3
	121–160	84	27.8
	≥ 161	67	22.1
	Missing	150	49.5
Median height (IQR) = 160 (165 -154.5) cm			
Systolic blood pressure (mmHg)**	< 120 (Normal)	60	19.8
	120 – 129 (Elevated)	39	12.9
	130 –139 (Stage1)	8	2.6
	≥ 140 (Stage 2)	12	4.0
	Missing	184	60.7
Median systolic blood pressure (IQR) = 117 (120 − 100) mmHg			
Diastolic blood pressure (mmHg)**	< 80 (Normal)*	68	22.5
	80 – 89 (Stage 1)	31	10.2
	≥ 90 (Stage 2)	20	6.6
	Missing	184	60.7
Median diastolic blood pressure (IQR) = 70 (180 − 60) mmHg			
Last CD4 count (cells/µL)	≤ 199	27	8.9
	200 – 499	79	26.1
	≥ 500	111	36.6
	Missing	86	28.4
Median CD4 count (IQR) = 538 (835 − 316) cells/µL			
Duration of HIV diagnosis (years)	1– 5	93	30.7
	6 – 10	155	51.1
	11–15	53	17.5
	≥ 16	2	0.7
Median duration of HIV diagnosis (IQR) = 7 (9 − 5) years			
Current HIV viral load (copies/ml)***	< 20 (Undetectable)	0	0.0
	< 1000 (Suppressed)	279	92.1
	> 1000 (Unsuppressed)	19	6.2
	Missing	5	1.7
Median current viral load (IQR) = 20 (57 − 20) copies/ml			

IQR = Interquartile range; * Diastolic blood pressure (BP) < 80 mmHg (Elevated) if Systolic BP = 120–129 (Elevated); **: American Heart Association [27]; ***WHO [28]

### Treatment characteristics of the study participants

More than half, (156/303, [51.5%]) of the SPs initiated ART 6–10 years prior to this study. As at the time of the study, most SPs (262/300, [86.5%]) were receiving TDF/3TC/DTG (TLD), and 77.6% (235/303) had completed the Isoniazid preventive therapy (IPT) as shown in Table 3. All SPs had received co-trimoxazole preventive therapy at one point or another prior to this study.

**Table 3:** Treatment characteristics of PLHIV on ART enrolled in the study (*n* = 303)

Variables	Responses	Frequency	(%)
Duration of ART on initiation (in years)	1 – 5	92	30.3
	6 – 10	156	51.5
	11–15	53	15.5
	≥ 16	2	0.7
Median duration of ART on initiation (IQR) = 7.0 (9 − 5) years			
Initial ART on diagnosis	TDF/3TC/EFV^1^	145	47.9
	AZT/3TC/NVP^2^	101	33.3
	TDF/3TC/DTG^3^	57	18.8
Current ART	TDF/3TC/DTG	262	86.5
	TDF/3TC/Lp/r^4^	41	13.5
Isoniazid Preventive Therapy	Not on therapy	18	5.9
	Currently on therapy	44	14.5
	Complete	235	77.6
	Incomplete	6	2.0

Key

IQR = Interquartile range,

^1^Tenofovir/Lamivudine/Efavirenz,

^2^Zidovudine/Lamivudine/Nevirapine,

^3^Tenofovir/Lamivudine/Dolutegravir,

^4^Tenofovir/Lamivudine/Lopinavir/ ritonavir

### Prevalence of HCV in PLHIV (HIV/HCV co-infection) in Ilorin, Nigeria

**Fig. 1:**
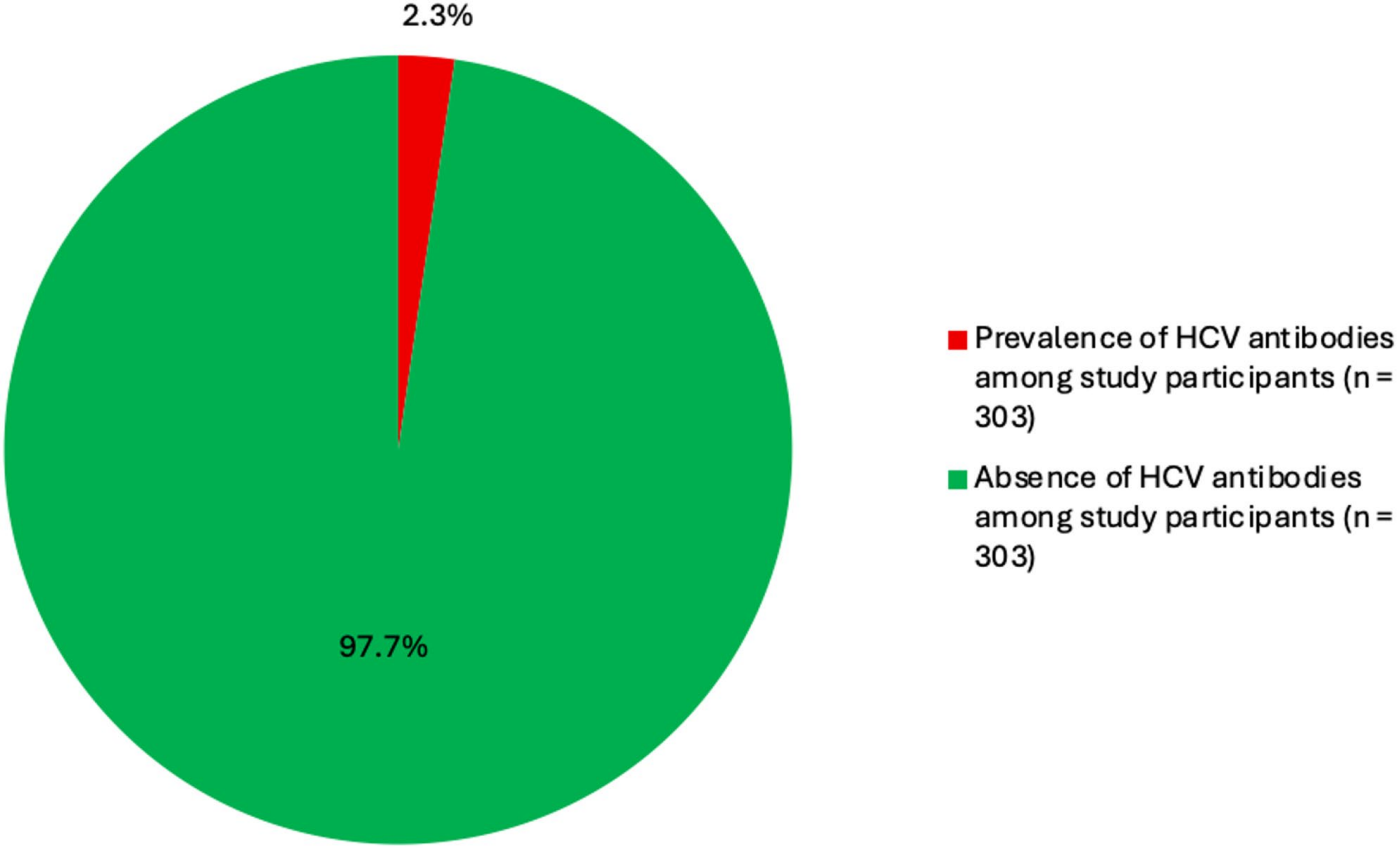
HCV antibody screening results of the study participants

Out of the three hundred and three (303) PLHIV that participated in the study, seven (7/303, [2.3%]) tested positive for the hepatitis C antibody screening as shown in Fig. 1. This gave a prevalence of 2.3% (95% CI, 0.9–4.7%).

### Association between demographic, clinical, and treatment variables and HIV/HCV co-infection

Bivariate analyses showed that viral load during ART at the time of the study was significantly associated with HIV/HCV co-infection (p = < 0.001). Most of the HIV/HCV co-infected PLHIV (6/7, [85.7%]) had unsuppressed viral load, during ART as at the time of the study (Table 4).

**Table 4:** Association between demographic variables, clinical Characteristics, treatment variables and HIV-HCV prevalence

Variables	HCV/HIC co-infected	HIV mono- infected	Fisher’s Exact Test	*P* - value
**Age**			0.127	1.000
≤ 40	4	149		
> 40	3	147		
**Sex**			1.957	0.533
Male	0	65	7.600	
Female	7	231		
**Educational qualification**			2.767	0.133
Senior secondary certificate	6	160		
Non-secondary certificate	1	137		
**Marital status**			1.185	0.599
Married	7	253		
Not married	0	43		
**Current Viral load (copies/ml)**			75.590	< 0.001*
≤ 1000 (Suppressed)	1	278		
> 1000(Unsuppressed)	6	13		
**Duration of HIV diagnosis (years)**			0.907	0.680
≤ 5	1	92		
> 5	6	204		
**Duration of ART on initiation (years**)			0.876	0.679
≤ 5	1	91		
> 5	6	205		
**Initial ART on diagnosis**			0.256	1.000
TDF-3TC-EFV	4	141		
AZT-3TC-NVP	2	99		
TDF-3TC-DTG	1	56		
**Current ART**			1.121	0.599
TDF-3TC-DTG	7	255		
TDF-3TC-Lp/	0	41		
**Isoniazid preventive therapy (IPT) status**			0.274	1.000
Complete	6	229		
Others**	1	67		

*: Significant association; ** Others: Incomplete IPT, on IPT, not on IPT

## Discussion

This study observed that HIV/HCV co-infection disproportionately affected young married women receiving ART in Ilorin, Nigeria. HIV/HCV co-infection was associated with viral load with more cases of HIV/HCV co-infections seen in the age range of 21–40 years. The observed HIV/HCV co-infection prevalence affecting more young women, corroborated findings of a global systematic review [2], but was lower than the observed prevalence in Bauchi, Nigeria [8], a Nigerian Teaching Hospital [11], Ituku Ozalla, Enugu, Nigeria [29], Brazil [30], Ethiopia [31], Malaysia [32] and global estimates [2, 3]. However, the observed prevalence is higher those reported Lagos [9, 10].

This co-infection is non-negligible, and requires special care, as it is known that HCV infection causes increased morbidity and mortality in PLHIV [8]. HIV/HCV co-infection in PLHIV on ART is of serious concern since it has impacts on the recovery of CD4 cells of the patients on ART [33]. It slows down CD4 recovery during ART compared to HIV mono-infection. This could be due to reduced thymic output and/or increased T cell activation [34]. Therefore, appropriate measures are required to ensure prompt diagnosis and reduced complications arising from HIV/HCV co-infection. Public health measures that emphasize preventive steps for HIV/HCV co-infection are primarily important for the control of the comorbidity.

The study observed that demographic variables did not show significant association with HIV/HCV co-infection. This is comparable to the findings reported in Lagos where there was no significant association between the investigated socio-demographic characteristics and HCV positivity [9]. However, other studies in Malaysia and Poland reported that HIV/HCV co-infection had a significantly higher prevalence in males [32, 35]. This contrast with regards to gender might be due to geographical variations of study setting and their associated risk factor profiles. Although there was no significant relationship between age and HCV positivity, more HIV/HCV positive cases were seen in the age range of 21–40, in this study. This is in contrast with findings from studies carried out in Poland and Nepal which identified significant relationships between age and HIV/HCV co-infection rate with the highest prevalence found in age groups 23–57 years and 30–39 years respectively [35, 36]. Similarly, age was significantly associated with HIV/HCV co-infection in other studies in Northern and Southern Nigeria [37, 38].

With reference to the clinical variables, this study found a significant association between HIV/HCV co-infection and HIV viral load of the study participants. This suggests that being positive for hepatitis C infection may influence HIV viral load. This could be due to the HIV-related immunosuppression. High viral load consequently leads to accelerated progression of damage to the liver. While this study did not examine the relationship between liver enzymes levels and viral load in HCV patients, an earlier study conducted in Tanzania suggested that liver enzyme abnormalities are significantly associated with HCV and higher in persons co-infected with HIV [39]. Hence, HIV/HCV co-infection is associated with higher rates of end-stage liver disease-related deaths when compared with HCV mono-infection [8]. This progression of liver damage in HIV/HCV-co-infected patients highlights the importance of early detection and prompt clinical care of patients.

No significant association was found between HIV/HCV status and the investigated treatment variables in this study. HIV/HCV co-infection possess shared mode of transmission which may be limited to lifestyle-related factors and exchange of body fluids, as such is unlikely related to the use of ART as observed in the study. However, all co-infected patients were currently receiving TLD. ART-related hepatotoxicity had been shown to be a relevant side effect in HIV/HCV co-infected patients [40]. Also, HCV treatment with Direct Acting Antiretrovirals in HIV/HCV co-infected patients resulted in more hepatic fibrosis compared to HCV mono-infection [41]. In addition, all the HIV/HCV co-infected patients were on co-trimoxazole preventive therapy while most of the patients had completed the isoniazid preventive therapy. Hepatotoxicity has been associated with co-trimoxazole, and isoniazid [42, 43]. These re-affirm the importance of determining the HCV status of diagnosed HIV patients before the initiation of ART and preventive therapies.

The study highlights objective findings that show the prevalence of HIV/HCV co-infection among PLHIV on ART and the associated patients’ demographic, clinical and treatment factors. However, this study is limited by the number of study sites (2 public hospitals), incomplete data due to some missing sociodemographic and clinical data from patients’ medical records and the exclusion of private hospitals. The exclusion of private healthcare settings in this study, possess a risk of selection bias, with the potential exclusion of relevant cases. Further, the adoption of convenient sampling approach posed a potential risk of limited representation which limits generalizability of study findings. This is because the sample was drawn from a subset of the population that was readily available which may not accurately reflect the characteristics of the entire population. Thus, the study participants do not accurately reflect the characteristics of the entire population. Additionally, the use of antibody testing for rapid HCV screening may have introduced false positives. It does not distinguish between active and past infection. The study did not include HCV RNA tests (polymerase chain reaction [PRC]) for confirmation, implying that the findings may not be absolute. Another limitation to this study is the non-availability of the specificity and sensitivity data of the Rapid Anti-HCV Test (Whole Blood/Serum/Plasma) by InTec Products Inc., China on the product label. The authors assumed high performance and compliance with rigorous benchmarks for product quality and efficacy.

## Conclusion

We found that HIV/HCV co-infection disproportionately affected young married women in this cohort of PLHIV on ART. Although the study was not completely representative of the population, the findings of this study underscore the importance of prioritizing the implementation of the National HIV Treatment guidelines in Nigeria on routine HCV screening among PLHIV before initiating ART. Also, hepatitis C status of PLHIV should be determined before initiation on preventive therapies. Longitudinal or multicentre studies should be conducted to strengthen evidence for national screening guidelines. Public awareness programs on HCV should be put in place to improve the awareness of PLHIV about the menace of hepatitis C, a preventable and completely treatable disease.

## Data Availability

Associated data is available from the corresponding author on reasonable request. The study participants’ privacy is to be protected in line with the European General Data Protection Regulation.
